# Supplementary material for: Opaque ontology: neuroimaging classification of ICD-10 diagnostic groups in the UK Biobank
Source: Gigascience. 2025 Feb 11;14:giae119. doi: 10.1093/gigascience/giae119 (PMC11811528; doi:10.1093/gigascience/giae119)
Supplement: giae119_Response_to_Reviewer_Comments_Original_Submission [file giae119_response_to_reviewer_comments_original_submission.pdf]

Author's Response To Reviewer Comments

Reviewer #1:
In this well-written and rigorous paper, Easley et al used the UK Biobank to investigate case-control classification accuracy in 17 ICD-10 diagnostic groups using structural MRI, functional MRI or socioeconomic feature sets. Only demyelinating diseases (using structural MRI) and depression (using functional MRI or socioeconomic data) could be classified above chance. Overall, it was a pleasure to read with a great collection of follow-up analyses, which has important implications for the field. I have a couple of suggestions that would strengthen the paper.

(1) I was left with a lingering question about comorbidity. A unique case list (with no comorbid individuals) was described and analysed but I have more broader questions surrounding comorbidity. What proportion of individuals have 1 or more diagnoses? How many individuals were shared across groups i.e. how similar was the depression group to the anxiety group? Were some diagnostic groups less comorbid than others? Basically, how fuzzy are the boundaries? If you were to run the same primary analysis (random forest classification) using only the unique case list, do you get the same results? Of course, I understand that this does not reflect reality as most individuals have comorbidities, but I am wondering if the algorithm performs better in this scenario given that many existing neuroimaging studies exclude individuals with comorbidities.

Thank you for the questions. Overall, 42.55% of individuals have more than one diagnosis across 17 diagnostic groups. A detailed breakdown of comorbidity rates has been added to the pre-existing demographics table in the revised manuscript in Table S1. Notably, 38.95% of individuals in the anxiety group overlap with the depression group. The newly added Figure S3 presents a heatmap that offers a broader insight into comorbidity patterns. This has been clarified in the revised manuscript on page 6:

As a result, we retained 17 ICD-10 diagnostic groups (see Table S1). The overall comorbidity rate, defined as the proportion of individuals who were in more than 1 of the 17 diagnostic groups, is 42.55%. Specifically, 38.95% of individuals in the anxiety group also have a diagnosis of depression, indicating significant overlap between these two diagnostic groups (see Figure S3). Thus, we generated a unique case list by excluding all individuals who were in more than one of the 17 diagnostic groups (Table S2). This unique case list was used for multiclass classification analysis.

As suggested by the reviewer, we performed the random forest classification on the unique list, it also shows the same results that the “Demyelinating diseases” ICD-10 diagnostic group (G35-37) was classified with the highest mean accuracy by both the surface-based structural neuroimaging features ($\mu=0.60$, $p=0.1896$) and volumetric features ($\mu=0.63$, $p=0.1896$). These results were not included in the revised manuscript for brevity, but we are happy to include them if the reviewer or editor prefers.

(2) The methods carefully describe how the control group was matched to each of the diagnostic groups, however I am curious about site-effects between scan sites. How did you control for site effects? As far as I understand, UK Biobank is a multisite dataset.

Thank you for pointing that out. We did not take site into account during the matching. In response to your comment, we performed statistical comparisons between sites and found that site differences were significant in 6 out of the 17 diagnostic groups. Detailed information on the Chi-square test is available in newly added Table S10. To control for potential site effects, we repeated the primary analysis using a leave-one-group-out cross-validation strategy, which ensures that individuals from the same site are not included in both the training and testing sets(REF). This approach prevents classification from being driven by site effects. The results are presented in the new Figure 1b, which demonstrate consistency with previous results suggesting that site effect did not impact the findings. The 'Demyelinating diseases' group continued to be classified above chance using both surface-based structural neuroimaging features and volumetric features. This has been added into the revised manuscripts on page 8:

To address site effects, the same primary classification was performed using leave-one-group-out cross-validation, and the results showed that the “Demyelinating diseases” ICD-10 diagnostic group (G35-37) was classified above chance (Fig. 1b).

(3) A suggestion to show which diagnoses are significant on the figures.

Thank you for your suggestion. Significant diagnoses are now labeled with asterisks on the top of the swarm plots in the revised manuscripts.

(4) I am not sure what the colour bar on Figure 2B means - Is it % of subjects?

Thank you for pointing that out. The color bar on the right side represents the number of individuals that fall into the intersection between the true and predicted diagnostic groups. The darker shades (near black) indicate fewer individuals, while the lighter shades (near white or light peach) indicate a higher number of individuals in that particular cell. This interpretation has been added to the revised manuscript in the caption of Figure 2:

Figure 2. (a) Size-corrected diagnostic classification based on surface-based structural neuroimaging features. Classification accuracy distributions across ICD-10 diagnostic groups matched in sample size. All classification results matched for sample size are reported in Table S7. (b) Size-corrected multiclass diagnostic classification. Confusion matrix of the multiclass classification for all 17 ICD-10 diagnostic groups along with healthy controls. The color bar on the right side represents the number of individuals that fall into the intersection between the true and predicted diagnostic groups. Darker shades (near black) indicate fewer individuals, while lighter shades (near white or light peach) indicate a higher number.

(5) Under the assumption that psychopathology lies on a continuum and that the diagnostic boundaries are somewhat arbitrary, I would be curious if you subsampled the most severe /"not unwell" for each diagnostic group, and compared to the "healthiest" controls, similar to how you subsampled for the young v old comparison.

Thank you for your suggestions. We agree that severe versus healthiest comparisons are of interest. Unfortunately, the UK Biobank does not contain severity information for many of the 17 diagnostic categories included in this work. As such, defining a consistent method for subsampling 'more severe' cases and the 'healthiest' controls is unfortunately not possible. This suggestion has been mentioned in the revised manuscripts on page 14:

Higher classification accuracy may be obtained by comparing severe patient groups to particularly healthy controls. Although this was not feasible based on available metrics in the UKB, it may be a fruitful area for future research.

(6) In the conclusion it states 'depression was significantly classified based on several functional neuroimaging feature sets but not based on structural feature sets' - I think it is also important to state depression was significantly classified based on socioeconomic factors. This work nicely aligns with a recent paper published by Winter et al, 2022 JAMA Psychiatry (doi:10.1001/jamapsychiatry.2022.1780)

Thank you for the suggestions. This has been added in the revised manuscripts on page 11:

After adding additional feature sets, the largest group (“Depression”, F32) was classified significantly above chance by both sociodemographic features [24] and several functional neuroimaging features, but not by structural neuroimaging features.

Reviewer #2:
The article presents a comprehensive investigation into the predictability of brain-related ICD categories from UK biobank data. It is an interesting study and will certainly provide food for thought. I do however have some concerns as well as questions and suggestions for the authors to consider.

Major concerns:
- The authors use only 'traditional' predictive models, even with the imaging data. However, traditional predictive models are not state of the art for medical imaging-based predictions. Rather, state of the art would be deep neural models such as visual transformers or convolutional neural networks. It is not clear to me to what extent the choice of predictive model and feature set (derived from images) could substantially impact the results of the study. By analogy, consider a hypothetical study in which natural language is modelled using a random forest. The predictive power would very likely be poor, but from that observation we would not infer that the natural language is not predictable, but rather that our choice of predictive model is not the right one for the task.

Thank you for this suggestion. One of our goals was to assess the predictivity of readily available imaging features and algorithms that have previously been found to correlate with the diseases of interest. However, we agree that there is scope for further work using state-of-the-art predictive models on imaging data directly, rather than on imaging-derived features as we do here. We have added a specific recommendation to the revised manuscript calling for future work in this direction (p. 14):

Second, although we tested our classifications across a range of feature sets and classifiers, it is possible that higher classification accuracy can be achieved using alternative feature sets or imaging modalities (e.g., clinically sensitive modalities such as susceptibility weighted imaging) and/or by using more sensitive classifier architectures; in particular, future work leveraging deep learning on image data may yield further insight.

- Following on from this point, the finding that the ICD-10 categories are not predictable has multiple potential explanations in the context of the study. (1) The ICD-10 categories are not coherent (they do not pick out a single phenotype set that can be learned by a classifier); (2) The brain is not the right place to look for the features corresponding to these conditions (e.g. if they are primarily metabolic/inflammation driven etc); or (3) The algorithms used were not good enough or the data were insufficient. The authors derive conclusion (1), but do not discuss the other possibilities.

We agree with the reviewer that multiple interpretations of our findings are possible. This has been clarified in the conclusion of the revised manuscript on page 15:

[...] unable to reliably classify 15 out of 17 ICD-10 diagnostic cases from matched controls. This classification failure implies that either (a) ICD-10 diagnostic categories in the UKB cannot be strongly predicted by neuroimaging data, suggesting limitations of the ICD-10 ontology, or (b) the extracted imaging features and/or predictive algorithms used were insufficient. Although future work should explore the predictive capabilities of imaging data (before feature extraction) with alternative algorithms, our findings demonstrate — at minimum — that standard predictive models using standard neuroimaging features do not predict ICD-10 labels in the largest epidemiological dataset available. These results reveal limits of the ICD-10 ontology. [...]

- page 19, second paragraph. "all variables in the mood & sentiment category and any variables related to smoking behaviors were excluded due to overlap with symptoms commonly observed" Not sure I understand this rationale for this exclusion given that these variables are likely to be predictive. Surely age, sex etc. are also commonly observed for several ICD diagnostic groups?

Thank you for this comment. To clarify, the variables in the mood and sentiment directly reflect key diagnostic symptoms of ICD-10 labels. For example, in order to receive an ICD-10 diagnosis of major

depressive disorder, patients have to meet 5 out of 9 criteria (including depressed mood, loss of interest, sleep disturbance, etc.). The variables that were excluded explicitly measured these key criteria. Similarly, tobacco and alcohol use data were excluded because 2 diagnostic groups (F10 and F17) are defined based on tobacco and alcohol use (Tables S1 and S2). Although the reviewer correctly notes that demographic variables may differ between ICD-10 diagnostic groups, the variables that were included (e.g., age, sex, etc.) do not directly reflect key diagnostic symptoms used for clinical diagnosis. This distinction was clarified in the revised manuscript on page 19:

Compared to prior work (see Appendix 2, Table S7 in [13]), all variables in the mood & sentiment category and any variables related to smoking behaviors were excluded because these variables directly reflect key diagnostic symptoms of ICD-10 labels. For example, to receive an ICD-10 diagnosis of major depressive disorder, patients have to meet 5 out of 9 criteria (including depressed mood, loss of interest, sleep disturbance, etc.). The variables that were excluded explicitly measured these key criteria.

Minor questions and suggestions:

- Why test prediction with either sociodemographic features or brain-derived features, but not both?

Thank you for the question. Although it may be of interest to perform a combined model in future work, we chose to keep the models separate to aid interpretability. Specifically, including both feature types in the model may lead to overfitting. Also, because sociodemographic features may be correlated with brain-derived features, testing separately can reduce confounding effects.

- On page 13, middle, Inequities in healthcare incentives are mentioned but these have not been introduced or discussed previously so their relevance to the current discussion point is not clear. Please elaborate.

We thank the reviewer for pointing out this lack of clarity. While a full analysis of the impact of sociodemographic stratification of healthcare access on diagnostic accuracies (especially along race and class lines) is well beyond the scope of this paper, we feel it is important to briefly acknowledge its potential importance as an obfuscating factor in diagnostic prediction. Furthermore, financial incentives can result in systematic misreporting of insurance diagnostic codes ("upcoding") that further problematize the relationship between neurological phenomena and ICD-10 diagnostic labels. Although our work does not have sufficient scope to disentangle the individual effects of these factors, they must be acknowledged as potential contributors to the suboptimality of ICD-10 labels as learning targets.

We have modified the text in the manuscript to clarify the specific challenges we wish to acknowledge:

Introduction (p. 5)
Third, our findings shed light on the limited validity of the ICD-10 diagnostic ontology, consistent with other research pointing to the limited reliability of diagnostic coding systems including the ICD-10 [8] and DSM-V [9,10] and their vulnerabilities to both systemic bias [11–16] and fraud [17–20]. Importantly, the use of suboptimal clinical labels as targets for machine learning models impedes meaningful biomarker discovery [21,22].

Discussion (p. 13)
In summary, our findings reveal limits of the ICD-10 diagnostic ontology that arise from a variety of complex sources, including clinical heterogeneity, lack of inter-rater reliability, and structural inequities in healthcare incentives and access.

References
11. Tait, R. C., Chibnall, J. T. & Kalauokalani, D. Provider judgments of patients in pain: seeking symptom

certainty. Pain Med. 10, 11–34 (2009).
12. Tversky, A. & Kahneman, D. Judgment under Uncertainty: Heuristics and Biases. Science 185, 1124–1131 (1974).
13. Bernheim, S. M., Ross, J. S., Krumholz, H. M. & Bradley, E. H. Influence of patients' socioeconomic status on clinical management decisions: a qualitative study. Ann. Fam. Med. 6, 53–59 (2008).
14. van Ryn, M. & Burke, J. The effect of patient race and socio-economic status on physicians' perceptions of patients. Soc. Sci. Med. 50, 813–828 (2000).
15. Job, C., Adenipekun, B., Cleves, A. & Samuriwo, R. Health professional's implicit bias of adult patients with low socioeconomic status (SES) and its effects on clinical decision-making: a scoping review protocol. BMJ Open 12, e059837 (2022).
16. Gara, M. A., Minsky, S., Silverstein, S. M., Miskimen, T. & Strakowski, S. M. A Naturalistic Study of Racial Disparities in Diagnoses at an Outpatient Behavioral Health Clinic. Psychiatr. Serv. 70, 130–134 (2019).
17. Geruso, M. & Layton, T. Upcoding: Evidence from Medicare on Squishy Risk Adjustment. J. Polit. Econ. 12, 984–1026 (2020).
18. Coustasse, A., Layton, W., Nelson, L. & Walker, V. UPCODING MEDICARE: IS HEALTHCARE FRAUD AND ABUSE INCREASING? Perspect. Health Inf. Manag. 18, 1f (2021).
19. Luo, W. & Gallagher, M. Unsupervised DRG Upcoding Detection in Healthcare Databases. in 2010 IEEE International Conference on Data Mining Workshops 600–605 (IEEE, 2010).
20. Moscelli, G. et al. Prospective payment systems and discretionary coding-Evidence from English mental health providers. Health Econ. 28, 387–402 (2019).
24. Winter, N. R. et al. Quantifying Deviations of Brain Structure and Function in Major Depressive Disorder Across Neuroimaging Modalities. JAMA Psychiatry 79, 879–888 (2022).
