# Supplementary material for: Opaque ontology: neuroimaging classification of ICD-10 diagnostic groups in the UK Biobank
Source: Gigascience. 2025 Feb 11;14:giae119. doi: 10.1093/gigascience/giae119 (PMC11811528; doi:10.1093/gigascience/giae119)
Supplement: giae119_Response_to_Reviewer_Comments_Revision_1 [file giae119_response_to_reviewer_comments_revision_1.pdf]

Author's Response To Reviewer Comments

Reviewer #2: The authors have, albeit minimally, addressed the points I raised in my previous review. Personally, I would prefer to see at least a little more exploration of the potential of more state of the art predictive approaches to change the substantial findings. However, the authors have now appropriately indicated this possibility in the interpretation of the findings.

Thank you for your feedback. We agree that exploring more state of the art predictive approaches is an important avenue for future research. While our current analysis provides a robust foundation for addressing our objectives, we recognize the potential for advanced methodologies to further refine or expand on our findings. We have emphasized this point in the discussion, highlighting it as a promising direction for future studies to build upon our work. We have added a specific recommendation to the revised manuscript calling for future work in this direction (p. 14):

Second, although we tested our classifications across a range of feature sets and classifiers, it is possible that higher classification accuracy can be achieved using alternative feature sets or imaging modalities (e.g., clinically sensitive modalities such as susceptibility weighted imaging) and/or by using more sensitive classifier architectures; in particular, future work leveraging deep learning on image data may yield further insight.
