# Supplementary material for: Opaque ontology: neuroimaging classification of ICD-10 diagnostic groups in the UK Biobank
Source: Gigascience. 2025 Feb 11;14:giae119. doi: 10.1093/gigascience/giae119 (PMC11811528; doi:10.1093/gigascience/giae119)
Supplement: giae119_Reviewer_1_Report_Original_Submission — Ashlea Segal -- 5/14/2024 [file giae119_reviewer_1_report_original_submission.pdf]

Reviewer Report

Title: Opaque Ontology: Neuroimaging Classification of ICD-10 Diagnostic Groups in the UK Biobank

Version: Original Submission **Date:** 5/14/2024

Reviewer name: Ashlea Segal

Reviewer Comments to Author:

In this well-written and rigorous paper, Easley et al used the UK Biobank to investigate case-control classification accuracy in 17 ICD-10 diagnostic groups using structural MRI, functional MRI or socioeconomic feature sets. Only demyelinating diseases (using structural MRI) and depression (using functional MRI or socioeconomic data) could be classified above chance. Overall, it was a pleasure to read with a great collection of follow-up analyses, which has important implications for the field. I have a couple of suggestions that would strengthen the paper.

(1) I was left with a lingering question about comorbidity. A unique case list (with no comorbid individuals) was described and analysed but I have more broader questions surrounding comorbidity. What proportion of individuals have a 1 or more diagnoses? How many individuals were shared across groups i.e. how similar was the depression group to the anxiety group? Were some diagnostic groups less comorbid than others? Basically, how fuzzy are the boundaries? If you were to run the same primary analysis (random forest classification) using only the unique case list, do you get the same results? Of course, I understand that this does not reflect reality as most individuals have comorbidities, but I am wondering if the algorithm performs better in this scenario given that many exiting neuroimaging studies exclude individuals with comorbidities.

(2) The methods carefully describe how the control group was matched to each of the diagnostic groups, however I am curious about site-effects between scan sites. How did you control for site effects? As far as I understand UK Biobank is a multisite dataset.

(3) A suggestion to show which diagnoses are significant on the figures.

(4) I am not sure what the colour bar on Figure 2B means - Is it % of subjects?

(5) Under the assumption that psychopathology lies on a continuum and that the diagnostic boundaries are somewhat arbitrary, I would be curious if you subsampled the most severe /"not unwell" for each diagnostic group, and compared to the "healthiest" controls, similar to how you subsampled for the young v old comparison.

(6) In the conclusion it states 'depression was significantly classified based on several functional neuroimaging feature sets but not based on structural feature sets' - I think it is also important to state depression was significantly classified based on socioeconomic factors. This work nicely aligns with a recent paper published by Winter et al, 2022 JAMA Psychiatry (doi:10.1001/jamapsychiatry.2022.1780)

Methods

Are the methods appropriate to the aims of the study, are they well described, and are necessary controls included? Choose an item.

Conclusions

Are the conclusions adequately supported by the data shown? Choose an item.

Reporting Standards

Does the manuscript adhere to the journal's guidelines on minimum standards of reporting? Choose an item.

Choose an item.

Statistics

Are you able to assess all statistics in the manuscript, including the appropriateness of statistical tests used? Choose an item.

Quality of Written English

Please indicate the quality of language in the manuscript: Choose an item.

Declaration of Competing Interests

Please complete a declaration of competing interests, considering the following questions:

- Have you in the past five years received reimbursements, fees, funding, or salary from an organisation that may in any way gain or lose financially from the publication of this manuscript, either now or in the future?
- Do you hold any stocks or shares in an organisation that may in any way gain or lose financially from the publication of this manuscript, either now or in the future?
- Do you hold or are you currently applying for any patents relating to the content of the manuscript?
- Have you received reimbursements, fees, funding, or salary from an organization that holds or has applied for patents relating to the content of the manuscript?
- Do you have any other financial competing interests?
- Do you have any non-financial competing interests in relation to this paper?

If you can answer no to all of the above, write 'I declare that I have no competing interests' below. If your reply is yes to any, please give details below.

I declare that I have no competing interest

I agree to the open peer review policy of the journal. I understand that my name will be included on my report to the authors and, if the manuscript is accepted for publication, my named report including any attachments I upload will be posted on the website along with the authors' responses. I agree for my

report to be made available under an Open Access Creative Commons CC-BY license (http://creativecommons.org/licenses/by/4.0/). I understand that any comments which I do not wish to be included in my named report can be included as confidential comments to the editors, which will not be published.

Choose an item.

To further support our reviewers, we have joined with Publons, where you can gain additional credit to further highlight your hard work (see: https://publons.com/journal/530/gigascience). On publication of this paper, your review will be automatically added to Publons, you can then choose whether or not to claim your Publons credit. I understand this statement.

Yes Choose an item.
