# Supplementary material for: Opaque ontology: neuroimaging classification of ICD-10 diagnostic groups in the UK Biobank
Source: Gigascience. 2025 Feb 11;14:giae119. doi: 10.1093/gigascience/giae119 (PMC11811528; doi:10.1093/gigascience/giae119)
Supplement: giae119_Reviewer_2_Report_Original_Submission — Janna Hastings -- 6/10/2024 [file giae119_reviewer_2_report_original_submission.pdf]

Reviewer Report

Title: Opaque Ontology: Neuroimaging Classification of ICD-10 Diagnostic Groups in the UK Biobank

Version: Original Submission **Date:** 6/10/2024

Reviewer name: Janna Hastings

Reviewer Comments to Author:

The article presents a comprehensive investigation into the predictability of brain-related ICD categories from UK biobank data. It is an interesting study and will certainly provide food for thought. I do however have some concerns as well as questions and suggestions for the authors to consider.
Major concerns:
- The authors use only 'traditional' predictive models, even with the imaging data. However, traditional predictive models are not state of the art for medical imaging-based predictions. Rather, state of the art would be deep neural models such as visual transformers or convolutional neural networks. It is not clear to me to what extent the choice of predictive model and feature set (derived from images) could substantially impact the results of the study. By analogy, consider a hypothetical study in which natural language is modelled using a random forest. The predictive power would very likely be poor, but from that observation we would not infer that the natural language is not predictable, but rather that our choice of predictive model is not the right one for the task.
- Following on from this point, the finding that the ICD-10 categories are not predictable has multiple potential explanations in the context of the study. (1) The ICD-10 categories are not coherent (they do not pick out a single phenotype set that can be learned by a classifier); (2) The brain is not the right place to look for the features corresponding to these conditions (e.g. if they are primarily metabolic/inflammation driven etc); or (3) The algorithms used were not good enough or the data were insufficient. The authors derive conclusion (1), but do not discuss the other possibilities.
- page 19, second paragraph. "all variables in the mood & sentiment category and any variables related to smoking behaviors were excluded due to overlap with symptoms commonly observed" Not sure I understand this rationale for this exclusion given that these variables are likely to be predictive. Surely age, sex etc. are also commonly observed for several ICD diagnostic groups?
Minor questions and suggestions:
- Why test prediction with either sociodemographic features or brain-derived features, but not both?
- On page 13, middle, Inequities in healthcare incentives are mentioned but these have not been introduced or discussed previously so their relevance to the current discussion point is not clear. Please elaborate.

Methods

Are the methods appropriate to the aims of the study, are they well described, and are necessary controls included? Choose an item.

Conclusions

Are the conclusions adequately supported by the data shown? Choose an item.

Reporting Standards

Does the manuscript adhere to the journal's guidelines on minimum standards of reporting? Choose an item.

Choose an item.

Statistics

Are you able to assess all statistics in the manuscript, including the appropriateness of statistical tests used? Choose an item.

Quality of Written English

Please indicate the quality of language in the manuscript: Choose an item.

Declaration of Competing Interests

Please complete a declaration of competing interests, considering the following questions:

- Have you in the past five years received reimbursements, fees, funding, or salary from an organisation that may in any way gain or lose financially from the publication of this manuscript, either now or in the future?
- Do you hold any stocks or shares in an organisation that may in any way gain or lose financially from the publication of this manuscript, either now or in the future?
- Do you hold or are you currently applying for any patents relating to the content of the manuscript?
- Have you received reimbursements, fees, funding, or salary from an organization that holds or has applied for patents relating to the content of the manuscript?
- Do you have any other financial competing interests?
- Do you have any non-financial competing interests in relation to this paper?

If you can answer no to all of the above, write 'I declare that I have no competing interests' below. If your reply is yes to any, please give details below.

I declare that I have no competing interests.

I agree to the open peer review policy of the journal. I understand that my name will be included on my report to the authors and, if the manuscript is accepted for publication, my named report including any attachments I upload will be posted on the website along with the authors' responses. I agree for my

report to be made available under an Open Access Creative Commons CC-BY license (http://creativecommons.org/licenses/by/4.0/). I understand that any comments which I do not wish to be included in my named report can be included as confidential comments to the editors, which will not be published.

Choose an item.

To further support our reviewers, we have joined with Publons, where you can gain additional credit to further highlight your hard work (see: https://publons.com/journal/530/gigascience). On publication of this paper, your review will be automatically added to Publons, you can then choose whether or not to claim your Publons credit. I understand this statement.

Yes Choose an item.
